# Perceived parental rearing styles and depression in Chinese adolescents: the mediating role of self-compassion

**DOI:** 10.3389/fpsyt.2024.1417355

**Published:** 2024-09-19

**Authors:** Yanzhen Ren, Shining Zhang, Caiying Huang, Jie Zhang, Tingyun Jiang, Yuan Fang

**Affiliations:** ^1^ Zhongshan Mental Health Center, The Third People’s Hospital of Zhongshan, Zhongshan, China; ^2^ Department of Medical, The Third People’s Hospital of Zhongshan, Zhongshan, China; ^3^ Department of Pediatric Psychology, The Third People’s Hospital of Zhongshan, Zhongshan, China

**Keywords:** perceived parental rearing styles, depression, self-compassion, adolescents, parental care, parental encouragement of autonomy, parental control

## Abstract

**Background:**

Adolescence is a period characterized by rapid biological and psychological change, and adolescents have a heightened risk of depression. Parental rearing is an important influencing factor for depression in adolescence. However, the mechanism of influence needs further exploration.

**Methods:**

A total of 1839 adolescents were recruited from a junior and a senior high school in Zhongshan City, Guangdong Province, China. They were requested to completed the Parental Bonding Instrument (PBI), Self-Compassion Scale (SCS), and Depression Anxiety and Stress Scale-21(DASS-21).

**Results:**

Adolescents recalled their mothers as being more caring and controlling than their fathers. Parental care (maternal care, paternal care) and parental encouragement of autonomy (maternal encouragement of autonomy, paternal encouragement of autonomy) were both negative predictors of depression, while parental control (maternal control, paternal control) was positive predictor of depression. Self-compassion mediated all relationships between parental rearing styles (parental care, parental encouragement of autonomy, and parental control) and depression but played different mediating roles (complete or incomplete mediating role) in different relationships.

**Conclusion:**

Self-compassion plays a mediator role in all relationships between perceived parental rearing styles (parental care, parental encouragement of autonomy, and parental control) and depression. Adolescents who grown up with less parental care, less parental encouragement of autonomy, and high parental control deserve special attention. Educators and clinicians could help those adolescents reduce the occurrence of depression by increasing their level of self-compassion.

## 1 Introduction

Depression is a major global public health problem. According to the World Health Organization (WHO), depression will become the world’s leading cause of disease and injury burden by 2030 (1). Among all ages, adolescents are particularly vulnerable to depression due to the multitude of rapid biological, psychological, and social changes that occur during this period (2). Previous studies have shown that the lifetime prevalence of depression among 13- to 18-year-olds is approximately 11% (3), with 14% of boys and 28% of girls reporting high and persistent depressive symptoms (4). Moreover, the onset of depression during adolescence can weaken a range of the social and cognitive abilities required to adapt to life problems, which in turn were highly predictive of prominent difficulties in youth, including poor educational achievement, physical ill health, non-suicidal self-injury (NSSI), and even suicide (5–7). Considering the high prevalence and costly ramifications of depressive symptoms, it is crucial to identify intervention targets that can be fostered and developed from a young age.

A variety of factors have been found to be associated with depressive symptoms in adolescents, among which family environmental factors such as parental rearing are always crucial factors for depression (8, 9). Parental rearing style refers to the means and methods taken by parents in the process of nurturing their children (10). In 1979, Parker divided this concept into three major factors: parental care, parental encouragement of autonomy and parental control (11). Extensive studies using different methods (e.g. cross-sectional studies, longitudinal studies) all seem to indicate that parental rearing style is essential to understand the origins of depression (12). Moreover, Studies have shown that parental care is more closely associated with depressive symptoms than parental encouraging autonomy or control (13, 14). With regard to parental care, research has consistently revealed that low parental care increases the risk of developing depression among children and adolescents (14–16). However, factors mediating or affecting these associations remain to be elucidated.

Self-compassion refers to the compassion and concern for oneself, which involves treating personal deficiencies and suffering with care and understanding, and recognizing that suffering affects everyone as part of the common human experience (17). As a form of self-view, self-compassion contains three components—self-kindness, the sense of common humanity, and mindfulness. Self-kindness entails treating oneself with a warm, supportive, and understanding attitude, especially when facing failure or difficulty; the sense of common humanity refers to recognizing that failure or difficulties are part of the human experience that everyone encounters; mindfulness involves being aware of one’s present experience of suffering with equanimity and balance (18).

Previous theories propose that interactions with significant others would help children develop an internal working model that contains their self-other representations (19). These self and self-other representations give rise to a set of attitudes and evaluations of themselves (20, 21). To put it simply, children often treat themselves and others as caregivers treated them (13). Neff and McGehee (22) proposed that self-compassion might represent an internalization of the parent-child relationship, children who grow up with more care and support are likely to develop higher levels of self-compassion. When children receive enough care or encouragement from their parents in early childhood, they internalize the positive information from their parents and tend to think of themselves as lovely and worthy of understanding and sympathy (22). As a result, their capacity for self-concern develops, and self-compassion may improve (23). In contrast, children who are raised in a neglectful, controlling, or indifferent way internalize negative information from caregivers and are expected to develop critical, negative attitudes toward themselves and exhibit low self-compassion (22, 24).

Furthermore, researchers have suggested that early experiences of care and warmth from parents might affect self-compassion through the self-soothing system (25–27). The soothing system evolved to tone down the threat system and signal to the organism that it is safe to rest and relax. This system can be fully developed in a warm and caring environment so that individuals can soothe themselves when facing difficulties and threats (28). When individuals fail to receive adequate care and warmth from their caregivers in childhood, they are prone to having an under-developed self-soothing system, and a reduced ability of self-compassion (29, 30). Consistent with these claim, empirical studies on adolescents and young adults have also indicated that self-compassion may originate from early interaction experiences with parents, and maternal support and healthy family functioning can predict higher levels of self-compassion (22). Specifically, individuals who perceived their parents were supportive, understanding and concerned, showed higher levels of self-compassion in their adulthoods (31). Conversely, individuals who experienced high control or emotional neglect in childhood show lower levels of self-compassion and are more prone to self-blame or neglect themselves (32, 33).

On the other hand, self-compassion is a protective factor against negative emotions (e.g. anxiety, depression) (34), and a lack of self-compassion may significantly contribute to the development and persistence of emotional difficulties in adolescents (35). Specifically, self-compassion enables individuals to cope with experiences of pain and failure in a kind and self-respecting manner, thereby freeing themselves from negative emotions (36, 37). Interestingly, among individuals who have high levels of fear related to becoming self-compassionate (that is, believing that they are so dreadful that they are undeserving of compassion or that it will lead to a drop in standards), negative emotions were found to be more severe and persistent (38). Consistent with these ideas, correlational and intervention studies have shown that self-compassion is strongly and negatively correlated with depression (39–41), and self-compassion based interventions have been found to decrease negative reactions to uncomfortable experiences, reduce self-criticism, and alleviate depression (35, 42).

Based on prior theories and studies, inappropriate parental rearing and aversive family environments (e.g. low early care) have been associated with lower levels of self-compassion, which in turn may contribute to the development of depression (17, 27). Thus, self-compassion may play a mediating role between parental rearing and depression. However, the mediating effect of self-compassion between parental rearing and depression has not yet been explored. To address this limitation, this study will examine the mechanism by which parental rearing leads to depression, and help to develop effective interventions.

In addition, previous studies have mainly examined the effects of overall parental rearing style on depression in adolescence but ignored the effects of rearing experiences with specific figures (paternal and maternal rearing) on depression. Furthermore, fathers and mothers play different roles in their children’s development (43, 44). It remains to be seen whether there are differences in the paternal rearing and maternal rearing perceived by children and whether they have different effects on children’s depression.

In summary, the present study was designed to explore the differences in paternal rearing and maternal rearing perceived by children among adolescents and to examine the mediating role of self-compassion between perceived parental rearing styles (paternal and maternal care, paternal and maternal encouragement of autonomy, paternal and maternal control) and depression among adolescents. Our hypotheses are as follows:

1. Adolescents perceive higher levels of care and control from their mothers compared to their fathers;
2. Different perceived parental rearing styles have different effects on adolescents’ depression, parental care (paternal care, maternal care) and parental encouragement of autonomy (paternal encouragement of autonomy, maternal encouragement of autonomy) have negative effects on adolescents’ depression, while parental control (paternal control, maternal control) has positive effect on depression;
3. Self-compassion will mediate all the relationships between perceived parental rearing styles (paternal and maternal care, paternal and maternal encouragement of autonomy, parental and maternal control) and depression.

The findings of this study will offer both theoretical and practical implications grounded in existing research. Theoretically, our results will provide evidence explaining how parental rearing influences adolescent depression and clarifying the role of self-compassion in this association. Practically, identifying how parental rearing impacts adolescent depression is crucial for developing effective prevention and intervention strategies for at-risk adolescents.

## 2 Method

### 2.1 Participants

The study enrolled participants from both a junior and a senior high school in Zhongshan City, Guangdong Province, China. These schools were chosen using a convenience sampling approach, and all the students within these schools were extended invitations to take part in the study. A total of 1973 adolescents, ranging from the first grade of junior high school to the third grade of high school, volunteered to participate between February and June 2023. After removing the invalid data (e.g. missing or contradictory data), a total of 1839 valid responses were collected, resulting in an effective response rate of 93.2%. The sample consisted of 953 (51.8%) boys and 886 (48.2%) girls aged between 12 and 18 years (mean= 15.07, SD= 1.52). Informed consent was obtained from all participants before the investigation. This study was approved by the Ethics Committee of The Third People’s Hospital of Zhongshan.

### 2.2 Procedure

Prior to beginning the questionnaires, a brief description of the study and its instruments was provided to inform and guide the participants through the survey. At a designated time, the participants were requested to complete a package of questionnaires (paper-and-pencil format) in quiet school classrooms.

### 2.3 Measures

#### 2.3.1 Parental bonding instrument

Parental Bonding Instrument (PBI) (11) was used to measure the impact of perceived parenting style during childhood on adolescents. This instrument is a self-reported questionnaire that retrospectively evaluates perceived parental style toward the child. The Chinese version of the PBI was used in this study, which consists of three dimensions: care, encouragement of autonomy and control (45). Items are rated on a 4-point likert scale ranging from 0 (very unlike) to 3 (very like), with higher scores indicating a stronger parenting attribute. Perceptions of bonding with mothers and fathers were measured. The Cronbach’s α of the maternal version (PBI-M) and the paternal version (PBI-F) in this study were 0.732 and 0.794, respectively.

#### 2.3.2 Self-compassion scale

The Self-Compassion Scale (SCS) compiled by Neff (46) and revised by Gong et al. (47) was used to measure the level of self-compassion in this study. It contains 12 items comprising 3 dimensions: self-kindness, the sense of common humanity, and mindfulness. Items are rated on a 5-point Likert scale ranging from 1 (almost never) to 5 (almost always), with higher scores indicating a higher level of self-compassion. The SCS has demonstrated good validity and reliability in Chinese adolescents and adults (45). In this study, the Cronbach’s α coefficient for the whole scale was 0.841.

#### 2.3.3 Depression anxiety and stress scale-21

The depression level was investigated using the depression subscale of Depression Anxiety and Stress Scale-21 (DASS-21) (48). The DASS-21 is a self-report assessment in which participants were asked to rate the extent to which certain experiences applied to them over the past week. The scale contains 21 items divided equally with 7 items into 3 subscales of stress, anxiety and depression. Items are scored on a 4-point Likert-type scale from 0(did not apply to me at all) to 3(applied to me most of the time). The Cronbach’s α coefficient of DASS-21depression subscale in this study was 0.851.

### 2.4 Data analyses

SPSS 24.0 was used to establish descriptive statistics on the data. The paired sample t-test was used to analyze the differences between paternal rearing and maternal rearing. The Pearson correlation coefficient was determined for correlation analysis between variables. Then, structural equation modeling (SEM) was used with Amos 24.0 software to analyze the mediating effects of self-compassion in the relationships between parental rearing styles (Parental care, encouragement of autonomy and control) and depression. The goodness of fit of the model was tested using the following fit indices (49, 50): Bentler comparative fit index (CFI) ≥ 0.90, Tucker-Lewis index (TLI) ≥ 0.90, root mean square error of approximation (RMSEA) <0.1, and standardized root mean residual (SRMR)< 0.08. We can conclude that the result of the SEM is acceptable and can be further analyzed. Finally, the bias-corrected bootstrap method (with 5000 resamples) and 95% confidence intervals (95% CI) were used for testing for mediating effects. A p<0.05 was considered statistically significant for all statistical tests.

## 3 Results

The scores of paternal care were significantly lower than those of maternal care (t=-18.245, p<0.001). The scores of paternal control were significantly lower than those of maternal control (t=-10.299, p<0.001). However, there were no significant differences between the scores of paternal encouragement of autonomy and maternal encouragement of autonomy (t=-1.180, p=0.238). Details are shown in **Table 1**.

**Table 1:** Comparison of paternal rearing and maternal rearing scores (N=1839).

		① Paternal	② Maternal	*t*	*p*
Care	M(SD)	23.43(6.61)	25.45(5.53)	-18.245	0.000^***^
Encouragement of autonomy	M(SD)	12.47(3.90)	12.54(3.78)	-1.180	0.238
Control	M(SD)	3.09(2.89)	3.57(3.13)	-10.299	0.000^***^

*p < 0.05; **p < 0.01; ***p < 0.001.

As shown in **Table 2**, depression was negatively correlated with self-compassion (r=-0.57, p <0.01), paternal care (r=-0.48, p<0.01) and maternal care (r=-0.47, p<0.01). Conversely, self-compassion was positively correlated with paternal care (r=0.48, p<0.01) and maternal care (r=0.46, p<0.01). Depression was negatively correlated with paternal encouragement of autonomy (r=-0.31, p<0.01) and maternal encouragement of autonomy (r=-0.29, p<0.01), while self-compassion was positively correlated with paternal encouragement of autonomy (r=0.39, p<0.01) and maternal encouragement of autonomy (r=0.37, p<0.01). Depression was positively correlated with paternal control (r=0.25, p<0.01) and maternal control (r=0.29, p<0.01), while self-compassion was negatively correlated with paternal control (r=-0.29, p<0.01) and maternal control (r=-0.31, p<0.01).

**Table 2:** Pearson’s r correlations between the variables.

Measures	M(SD)	(2)	(3)	(4)	(5)	(6)	(7)	(8)
(1) Paternal care	23.43(6.61)	0.71^**^	0.57^**^	0.41^**^	-0.39^**^	-0.38^**^	0.48^**^	-0.48^**^
(2) Maternal care	25.45(5.53)		0.48^**^	0.57^**^	-0.38^**^	-0.43^**^	0.46^**^	-0.47^**^
(3) Paternal encouragement of autonomy	12.47(3.90)			0.79^**^	-0.42^**^	-0.32^**^	0.39^**^	-0.31^**^
(4) Maternal encouragement of autonomy	12.54(3.78)				-0.34^**^	-0.41^**^	0.37^**^	-0.29^**^
(5) Paternal control	3.09(2.89)					0.78^**^	-0.29^**^	0.25^**^
(6) Maternal control	3.57(3.13)						-0.31^**^	0.29^**^
(7) SCS total score	40.46(8.54)							-0.57^**^
(8) DASS-21 Depression	6.48(7.33)							

SCS, Self-Compassion Scale; DASS-21, Depression Anxiety and Stress Scale-21.

*p < 0.05; **p < 0.01.

**Figure 1:**
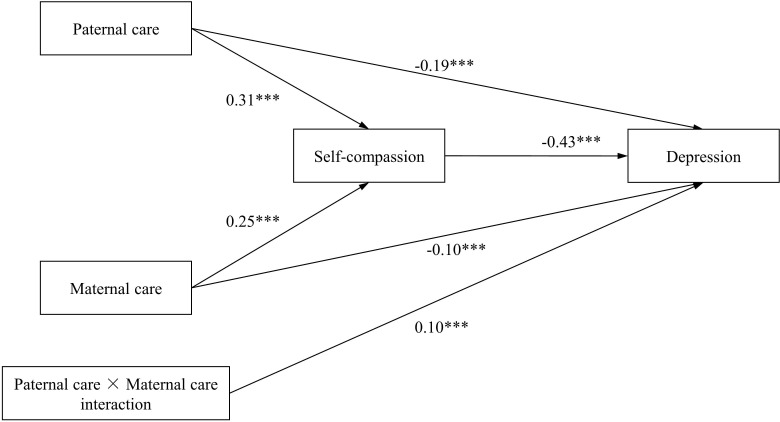
Path diagram with structural equation modeling results and standardized path coefficients (model 1). *p < 0.05; **p < 0.01 ; ***p < 0.001.

We first verified the mediating role of self-compassion in the influence of paternal care and maternal care on depression (model 1). The hypothesized model demonstrated a good fit of the data (χ^2^/df=9.525, p=0.002, RMSEA=0.068, CFI=0.997, TLI=0.972, SRMR=0.016). As shown in **Figure 1**, all pathways in the model were significant. Paternal care and maternal care positively predict self-compassion (β=0.31, p<0.001; β=0.25, p<0.001), which in turn negatively predicts depression (β=-0.43, p<0.001). The direct effects of paternal care and maternal care on depression were still significant when controlling for self-compassion (β=-0.19 and β=-0.10, respectively; ps<0.001). Therefore, self-compassion plays a partial mediating role between these relationships. Furthermore, the bootstrap analyses showed that the relationship between paternal care and depression as well as the relationship between maternal care and depression were both significantly mediated by self-compassion (the indirect effect =-0.131, 95% CI [-0.159, -0.106]; the indirect effect=-0.105, 95%CI [-0.134, -0.078], respectively) (see **Table 3**).

**Table 3:** The paths and effect analysis.

Effect	Paths	Effect size	95% CI
Direct effect	Paternal care→ Depression	-0.191	-0.255- -0.132
Indirect effect	Paternal care→ Self-compassion →Depression	-0.131	-0.159- -0.106
Direct effect	Maternal care→ Depression	-0.100	-0.156- -0.042
Indirect effect	Maternal care→ Self-compassion →Depression	-0.105	-0.134- -0.078
Direct effect	Paternal encouragement of autonomy→ Depression	-0.054	-0.122- 0.009
Indirect effect	Paternal encouragement of autonomy→ Self-compassion →Depression	-0.144	-0.181- -0.110
Direct effect	Maternal encouragement of autonomy→ Depression	-0.049	-0.111- 0.019
Indirect effect	Maternal encouragement of autonomy→ Self-compassion →Depression	-0.081	-0.118- -0.043
Direct effect	Paternal control→ Depression	-0.027	-0.102- 0.050
Indirect effect	Paternal control→ Self-compassion →Depression	0.065	0.029- 0.103
Direct effect	Maternal control→ Depression	0.131	0.059- 0.205
Indirect effect	Maternal control→ Self-compassion →Depression	0.117	0.076 -0.157

**Figure 2:**
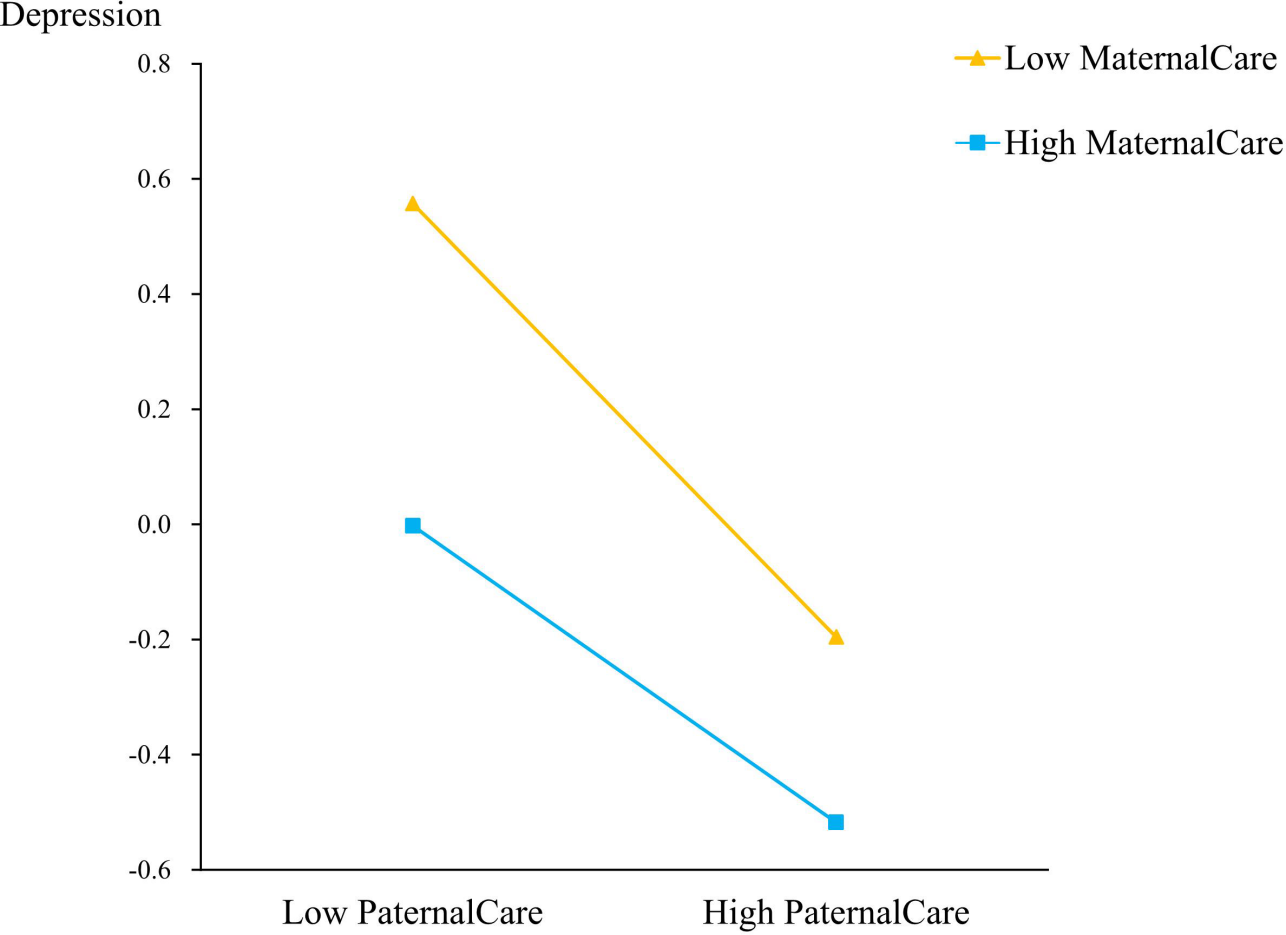
Moderation effect of Maternal care on the relation between Paternal care and Depression. High and low values are 1 standard deviation above and below the mean, respectively.

The interaction of paternal care and maternal care predicting depression was significant (β = 0.10, p<0.001). To examine the interaction between paternal care and maternal care on depression, simple slope analyses were utilized to demonstrate whether paternal care has a significant effect on depression at low(-1SD) and high (+1SD) levels of maternal care. For those with low levels of maternal care, higher paternal care was associated with lower depression; the simple slope was -0.274 (p<0.001). In addition, the effect of paternal care on depression was also significant when maternal care was high, that is, for those with high levels of maternal care, higher paternal care was associated with lower depression. The simple slope was -0.110 (p<0.01). Although both those with either low or high on the maternal care showed high depression when paternal care was low, the slope in those with low maternal care was significantly steeper (becomes more strongly negative as levels of maternal care decrease, as shown by the significant interaction) than in those with high maternal care. In other words, maternal care mitigated the depression shown in those with low paternal care (see **Figure 2**).

**Figure 3:**
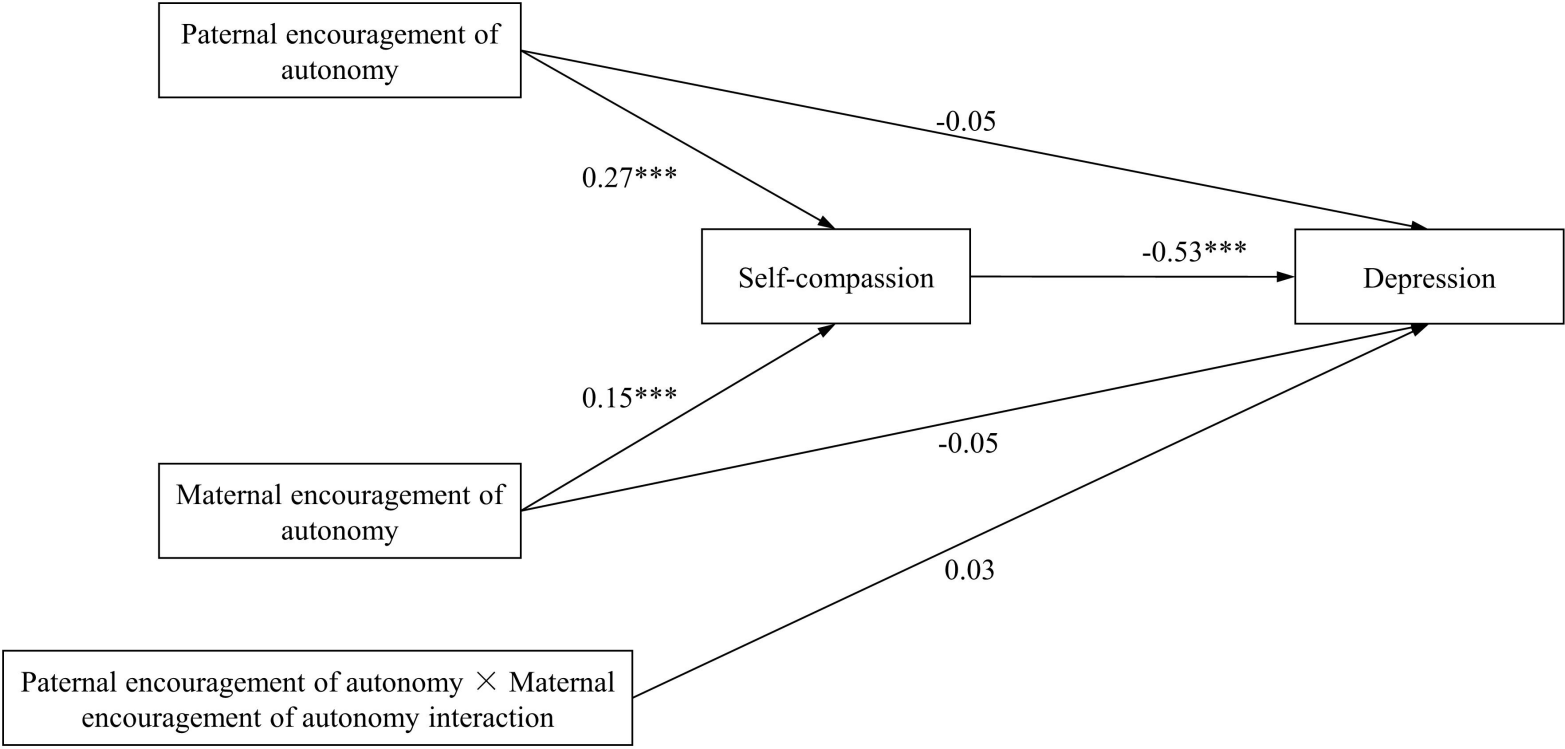
Path diagram with structural equation modeling results and standardized path coefficients (model 2). *p < 0.05; **p < 0.01; ***p < 0.001.

**Figure 4:**
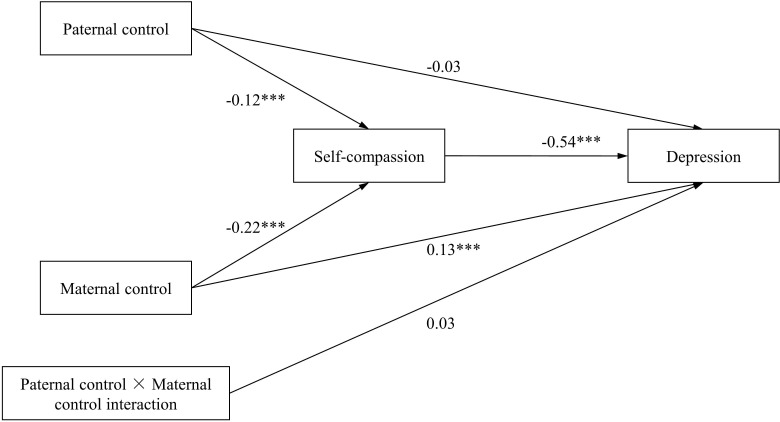
Path diagram with structural equation modeling results and standardized path coefficients in (model 3). *p < 0.05; **p < 0.01; ***p < 0.001.

We further explored the mediating effects of self-compassion in the influence of paternal encouragement of autonomy and maternal encouragement of autonomy on depression (model 2), as well as the mediating effects of self-compassion in the influence of paternal control and maternal control on depression (model 3). The hypothesized models both demonstrated an adequate fit of the data (model 2: χ^2^/df=9.040, p=0.003, RMSEA=0.066, CFI=0.997, TLI=0.973, SRMR=0.018; model3: χ^2^/df=13.731, p=0.000, RMSEA=0.083, CFI=0.996, TLI=0.961, SRMR=0.021). In model 2, both paternal encouragement of autonomy and maternal encouragement of autonomy positively predicts self-compassion (β=0.27 and β=0.15, respectively; ps<0.001), and self-compassion negatively predicts depression (β=-0.53, p<0.001). The mediating effects of self-compassion between paternal encouragement of autonomy as well as maternal encouragement of autonomy and depression were both significant (with the indirect effect =-0.144, 95% CI[-0.181, -0.110]; the indirect effect =-0.081, 95% CI[-0.118, -0.043]) (see **Table 3**). The direct effects of paternal encouragement of autonomy and maternal encouragement of autonomy on depression were not significant (β=-0.05, p=0.08; β=-0.05, p=0.12) (see **Figure 3**). Therefore, self-compassion plays the complete mediating role in the relationships between paternal encouragement of autonomy as well as maternal encouragement of autonomy and depression. In addition, the interaction of paternal encouragement of autonomy and maternal encouragement of autonomy predicting depression was not significant (β=0.03, p=0.12). In model 3, both paternal control and maternal control negatively predicts self-compassion (β=-0.12 and β=-0.22, respectively; ps<0.001), and self-compassion negatively predicts depression (β=-0.54, p<0.001). The mediating effects of self-compassion between paternal control as well as maternal control and depression were both significant (the indirect effect =0.065, 95% CI[0.029, 0.103]; the indirect effect =0.117, 95% CI[0.076, 0.157]) (see **Table 3**). The direct effect of paternal control on depression was not significant (β=-0.03, p=0.40), but the direct effect of maternal control on depression was still significant (β=0.13, p<0.001) (see **Figure 4**). Therefore, self-compassion plays a complete mediating role between paternal control and depression, but plays an incomplete mediating role between maternal control and depression. In addition, the interaction of paternal control and maternal control predicting depression was not significant (β=0.03, p=0.15).

## 4 Discussion

The present study aimed to explore the differences in paternal rearing and maternal rearing perceived by children among adolescents and examine the mediating role of self-compassion in the relationships between perceived parental rearing (paternal and maternal care, paternal and maternal encouragement of autonomy, paternal and maternal control) and depression during adolescence. The results revealed that adolescents recalled their mothers as more caring and controlling compared to their fathers. The main finding of this study was that self-compassion mediated all relationships between parental rearing styles (parental care, parental encouragement of autonomy, and parental control) and depression, but played different mediating roles (complete or incomplete) in different relationships. This indicates that different perceived parental rearing styles have distinct mechanisms of influence on adolescent depression. Specifically, self-compassion partially mediated the relationship between both paternal care and depression, and maternal care and depression. This suggests that both paternal and maternal care have a direct impact on adolescent depression, as well as an indirect impact through self-compassion. However, self-compassion fully mediated the relationship between paternal encouragement of autonomy and depression, as well as between maternal encouragement of autonomy and depression, indicating that both paternal and maternal encouragement of autonomy influence depression entirely through self-compassion. Furthermore, self-compassion fully mediated the relationship between paternal control and depression, but only partially mediated the relationship between maternal control and depression. This implies that paternal control affects depression entirely through self-compassion, while maternal control can not only directly affect depression, but also indirectly affect depression through self-compassion.

In our research, adolescents recalled their mothers as more caring and controlling than their fathers, which was consistent with the results of previous studies (51, 52). This may be due to the following three reasons: firstly, from the perspective of biology, the connection and attachment between the child and the mother should be closer (20); Secondly, from the perspective of household labor division, compared to the responsibility of earning money from the father, the mother assumed more responsibility for taking care of the family and child, and thus showed more care and higher control to the child (worrying about the danger of the child) (53, 54); Thirdly, from the perspective of emotional expression, the father’s way of expressing emotions is more restrained, while the mother’s way of expressing emotions is more open and direct, resulting in children more easily to feel the care and control from the mother (55, 56).

The mediation effect analysis results showed that self-compassion partially mediated the effects of paternal care and maternal care on depression in adolescents. On the one hand, both paternal care and maternal care have negative direct effects on depression, which is consistent with the previous studies that showed the ill effects of low parental care on child depression (12, 57). Parents play a crucial role in an individual’s growth, and their care and emotional support are vital and irreplaceable. Individuals who receive less care and support from their parents find it difficult to cope with stress and are prone to depression. On the other hand, both paternal care and maternal care can indirectly affect the depression through self-compassion, suggesting that less parental care is detrimental to the development of an adolescent’s self-compassion ability and thus increases the likelihood of depression.

The Gilbert’s theory of compassion (26) points out that when individuals fail to receive adequate care and warmth from their caregivers in childhood, they are prone to having an under-developed self-soothing system, which makes individuals less likely to face difficulties in a self-compassionate way. In addition, adolescents with low parental care may internalize and migrate how their parents treat them to how they treat themselves (20, 22). Specifically, when living with low parental care, adolescents may often internalize this as their own problem and see themselves as being unworthy of love (58), thereby decreasing the level of self-compassion. After all, if individuals do not grow up feeling warm and cared for, it might be foreign, challenging, and even frightening to try and comfort themselves with compassion (59). A large number of empirical studies have also shown that individuals who have experienced less parental care in childhood show lower levels of self-compassion in adolescents (60, 61). Individuals with low self-compassion are more prone to treating themselves unkindly, adopting a self-critical, depressive and intolerant attitude towards stressful events. When facing suffering, they tend to think that they are isolated, and that suffering is unbearable. They tend to engage in thinking inhibition and are unable to face the stressful events directly, which makes them more prone to depression (62). A large number of empirical studies have also confirmed a negative association between self-compassion and depressive symptoms (41, 63–66).

This study found that paternal care and maternal care have an interactive effect on depression. Specifically, the relationship between lower paternal care and higher depression was mitigated by maternal care, such that at higher levels of maternal care, this association was weaker. This is consistent with prior studies (67), which suggest that children’s depression is not only the result of one parent, but the result of the common influence of both parents. Generally, adolescents experience less paternal care, which can lead to a higher level of depression. However, if adolescents with little paternal care encounter a high level of maternal care, their depression will still be at a relatively low level.

The mediation effect analysis results showed that self-compassion completely mediated the effect of paternal control on depression, but partially mediated the effect of maternal control on depression. These findings suggest that both paternal control and maternal control are detrimental to the development of adolescents’ self-compassion ability, thereby increasing the likelihood of depression. Additionally, maternal control has a direct effect on depression in adolescents. Adolescents living under high parental control often prioritize their parents’ wishes and needs, which can prevent them from acting according to their own desires (68). Over time, adolescents may feel that their ideas and wishes are unimportant, breeding a sense of disgust for themselves and life, and easy to fall into a passive depressive state. Numerous prior studies have consistently shown a positive association between psychological control and depression (69–71). According to the self-determination theory (SDT), parental psychological control restricts and violates children’s basic psychological needs for autonomy, thwarts their autonomy and threatens their emerging sense of self (71–73). That is, high parental control makes children fall into self-doubt and perceive that they are unable to manage themselves, incompetent and need to be controlled. When faced with difficulties, they are more likely to resort to self-criticism rather than self-compassion, making them more susceptible to depression. Previous studies have also found that high parental control leads to self-depreciation and reduces the level of self-compassion in children (33, 74). On the contrary, parental encouragement of autonomy can promote the development of adolescents’ autonomy and independence, and is conducive to adolescents’ self-affirmation and self-compassion. This, in turn, reduces the risk of adolescent depression. As this study found, self-compassion completely mediated the effects of paternal encouragement of autonomy and maternal encouragement of autonomy on depression.

There are some limitations to this study. First, this study used a cross-sectional design, which makes it unable to demonstrate a true causal relationship. Therefore, longitudinal studies are greatly needed to verify the causal hypothesis. Second, the use of convenience sampling limits the generalizability of the findings to adolescents across different institutions and geographic regions. Further studies involving students from various secondary schools and regions are warranted. Third, the statistical effects might have been affected by the participant bias due to the retrospective self-reported nature of the survey (e.g., The Parental Bonding Instrument), Future studies are encouraged to use a combination of self-reported questionnaires and parent-reported questionnaires to collect relevant data. Fourth, this study cannot exclude the possibility that the findings were influenced by other variables not explored here (e.g. sex, family economic status), which requires further research to enhance the clarity and robustness of this study. Finally, the relationship among parental rearing, various dimensions of self-compassion and depression has not been studied in detail, and it is unclear whether these relationships are consistent across different left-behind experience groups, which provide directions for further research.

## 5 Conclusion

In conclusion, this study showed that parental rearing in childhood and self-compassion influence depression in adolescents, and that self-compassion plays a mediating role between all parental rearing styles (parental care, parental encouragement of autonomy, and parental control) and depression. These findings reveal the possible potential mechanism by which parental rearing may influence adolescent depression and provide preliminary empirical support for the implementation of self-compassion in the prevention and intervention of adolescent depression. For children, caregivers should give enough care and encouragement of autonomy but less control, which could help them develop self-compassion and reduce the probability of depression in adolescence. For depressed adolescents, clinicians can conduct self-compassion based group interventions help them cultivate self-compassion, and reduce the level of depression.

## Data Availability

The raw data supporting the conclusions of this article will be made available by the authors, without undue reservation.
